# Serum Biomarkers for Inflammatory Bowel Disease

**DOI:** 10.3389/fmed.2020.00123

**Published:** 2020-04-22

**Authors:** Peng Chen, Gaoshi Zhou, Jingxia Lin, Li Li, Zhirong Zeng, Minhu Chen, Shenghong Zhang

**Affiliations:** ^1^Division of Gastroenterology, The First Affiliated Hospital, Sun Yat-sen University, Guangzhou, China; ^2^Division of Blood Transfusion, The First Affiliated Hospital, Sun Yat-sen University, Guangzhou, China

**Keywords:** Inflammatory bowel disease, Serum biomarker, C-reactive protein, non-coding RNA, diagnosis, activity evaluation, prognosis prediction

## Abstract

**Background:** Inflammatory bowel disease (IBD), including Crohn's disease and ulcerative colitis, is a chronic, inflammatory disorder of the gastrointestinal tract. As the novel therapeutic goal and biologicals are widely recognized, accurate assessment of disease and prediction of therapeutic response have become a crucial challenge in clinical practice. Also, because of the continuously rising incidence, convenient and economical methods of diagnosis and clinical assessment are urgently needed. Recently, serum biomarkers have made a great progress and become a focus in IBD study because they are non-invasive, convenient, and relatively inexpensive than are markers in biopsy tissue, stool, breath, and other body fluids.

**Aims:** To review the available data on serological biomarkers for IBD.

**Methods:** We searched PubMed using predefined key words on relevant literatures of serum biomarkers regarding diagnosis, evaluation of therapeutic efficacy, surveillance of disease activity, and assessment of prognosis for IBD.

**Results:** We reviewed serological biomarkers that are well-established and widely used (e.g., C-reactive protein), newly discovered biomarkers (e.g., cytokines, antibodies, and non-coding RNAs), and also recently advancements in serological biomarkers (e.g., metabolomics and proteomics) that are used in different aspects of IBD management.

**Conclusions:** With such a wealth of researches, to date, there are still no ideal serum biomarkers for IBD. Serum profiling and non-coding RNAs are just starting to blossom but reveal great promise for future clinical practice. Combining different biomarkers can be valuable in improving performance of disease evaluation.

## Introduction

Inflammatory bowel disease (IBD), including Crohn's disease (CD) and ulcerative colitis (UC), is a lifelong disease with symptoms that tend to wax and wane and frequent exacerbations. The incidence of IBD is increasing steadily and has become an important public health issue in Western countries and newly industrialized countries (1). Our group reported that 32.9% of patients with CD eventually become disabled by the disease (2). Between 5 and 15% of UC patients required colectomy, and 80% of CD patients need at least one operation in their lifetime, which markedly affects quality of life (3). The key to improve the prognosis of patients with IBD is strict and effective management.

After IBD is diagnosed, determination of the subclassification of IBD is sometimes difficult (4). It is also a challenge to differentiate between IBD and colitis of other etiologies. An inaccurate diagnosis can have an adverse impact on treatment effectiveness and future management. Treatment decisions also depend on accurate assessment of disease severity. Adequate monitoring is crucial for identifying disease relapse and administering timely treatments. Treatments should be individualized and adjusted during the disease course on the basis of disease severity, extent of lesions, disease behavior, and responses to drugs. Responses to treatments must be closely monitored to determine effectiveness and avoid disease complications. Studies of patients with CD have shown that mucosal healing increases steroid-free remission rates and reduces the risk of re-hospitalizations and surgery (5, 6).

In patients with IBD, clinical symptoms are not often consistent with disease activity. It is difficult to distinguish between functional bowel symptoms and those of active disease. Patients with mucosal lesions may not have clinical symptoms, or only present with mild symptoms (7). Objective measurements are required to assess and monitor IBD disease activity. However, there is no single “gold standard” test for diagnosing IBD, assessing disease severity, or evaluating the response to treatment. Physicians rely on a combination of clinical symptoms, laboratory indices, radiological investigations, endoscopy, and histological examination of tissue specimens to assess disease activity and make treatment decisions (8).

Endoscopy and histological evaluations of tissue specimens are required to be able diagnose IBD, and endoscopy offers real-time imaging of mucosal lesions. However, it is uncomfortable, time-consuming, costly, and accompanied by a risk of perforation. Additionally, endoscopy cannot assess transmural inflammation. Patients with transmural healing have been shown to have improved long-term outcomes than have patients who have mucosal healing but with magnetic resonance enterography active disease (9).

So far, the treatment of IBD includes induction therapy and maintenance therapy (10, 11). Traditional therapeutic drugs include amino salicylates, glucocorticoid (GC), immunosuppressive (such as azathioprine methotrexate), and TNF-α monoclonal antibodies (12). Despite that multiple drugs are available for treatment of IBD, a large proportion of patients either have no response or lose response to therapy. Approximately 30% of CD patients fail to respond to infliximab, and the annual risk of losing response to infliximab is about 13% per patient year (13–15). For the past few years, advances in novel IBD treatments have provided new options for these patients. Gut-specific-α4β7 integrin antibody (vedolizumab and etrolizumab) and IL-12/IL-23 inhibitors (ustekinumab and risankizumab) have shown therapeutic effect to patients who have refractory IBD or lose response to anti-TNF treatment (16–18). As more and more new therapies are put in clinical practice, precision medicine will become a vital challenge for IBD treatment. This involves accurate stratification of patients, the use of effective and reliable biomarkers, and the determination of the optimal clinical pathways for different individuals (19).

Various biomarkers for IBD have been studied over the past decades, and some of them are widely used in clinical practice. An ideal biomarker should be non-invasive, sensitive, disease specific, easy to perform, and cost-effective (20). To date, there is no ideal biomarker that possesses all the aforementioned qualities to accurately diagnose IBD, to differentiate between subtypes of IBD, or to monitor disease activity. IBD biomarkers have been identified in colonic tissue, blood, stool, urine, and breath. Blood-based biomarkers are non-invasive, can be readily obtained, are not easily contaminated, and are the most widely used.

**Figure 1:**
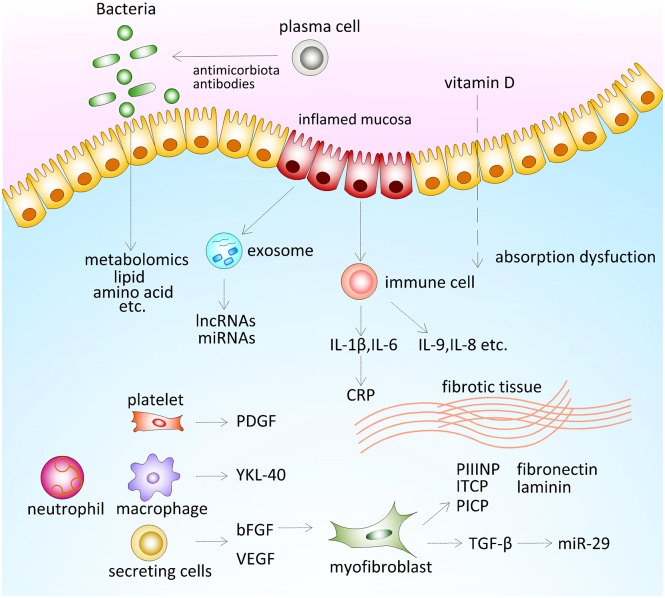
Serum biomarkers for inflammatory bowel disease (IBD) management. Antimicrobial antibodies are antibodies that targeted microbiota-derived antigens through the interplay between host immune system and gut microbiota. Both environmental factors and gut microbiota influence metabolome of patients. Patients with IBD tend to show a low level of vitamin D, which is partly caused by absorption dysfunction due to active disease. Intestinal epithelial cells from the inflamed mucosa secrete exosome, which contains microRNA (miRNAs) and long non-coding RNAs (lncRNAs) and other functional proteins in circulation. Pro-inflammatory cytokines were secreted by activated immune cells, which induce the expression of CRP. Excessive deposition of extracellular matrix (ECM) components include laminin, fibronectin, collagen, and its propeptide. Several growth factors mediating development of fibrostenosis [platelet-derived growth factor (PDGF), basic fibroblast growth factor (bFGF), vascular endothelial growth factor (VEGF), and YKL-40]. bFGF promotes tissue healing by regulating proliferation of myofibroblast. Transforming growth factor (TGF)-β induced the expression of collagen and promoted intestinal fibrosis through inhibiting the expression of miR-29.

The purpose of this article is to review serological biomarkers used in IBD management, from classical biomarkers, which are well-established and widely used, to new, novel, and promising markers. The use of biomarkers for the diagnosis and classification of IBD, surveillance of disease activity, predicting and monitoring treatment effectiveness, and determining a prognosis are discussed. Because intestinal fibrosis notably affects prognosis in IBD patients, serum markers for intestinal fibrosis will be discussed in this article (Table 1, Figure 1).

**Table 1:** Serum biomarkers for IBD.

**Biomarker**		**Association**
Antibodies	pANCA	IBD subclassification (UC-specificity), lower response rate to IFX therapy (21, 22)
	ASCA	IBD subclassification (CD-specificity), early disease onset, fibrostenosing behavior, internal-penetrating disease behavior (23)
	Anti-GP2	IBD subclassification (CD patients with ileum involvement) (24, 25)
	Anti-CUZD1	CD patients with stricturing behavior (26)
	Anti-CHI3L1	IBD subclassification (CD patients) (27)
	Anti-GM-CSF	IBD subclassification (CD patients), aggressive disease, ileal involvement (28)
	Anti-ACA	Diagnostic potential (29)
	Anti-PS/PT	Diagnostic potential (29)
	ALCA	IBD subclassification (CD patients) (30)
	ACCA	IBD subclassification (CD), steroid dependency (30, 31)
	AMCA	IBD subclassification (CD) (30)
	Anti-OmpC	IBD subclassification (CD-specificity), repeated surgeries, poor clinical response, early postoperative recurrence (32–35)
	Anti-I2	IBD subclassification (CD-specificity), stricturing behavior, longer disease duration, and early postoperative recurrence (32, 33, 36, 37)
	Anti-CBir1	IBD subclassification (CD-specificity), stricturing behavior, penetrating behavior, longer disease duration, early postoperative recurrence (32, 33, 36, 37)
	Anti-L	IBD subclassification (CD-specificity), penetrating behavior, surgery (38)
	Anti-C	IBD subclassification (CD-specificity), penetrating behavior, the need for surgery (38)
	Anti-IFI16	Predicting clinical response (39)
CRP		Surveillance of disease activity, indicator of active disease, predicting clinical response (40, 41)
LL-37		Surveillance of disease activity, stricture disease in CD patients (42)
TFF3		Surveillance of disease activity (43)
Cytokines	IL-1β, IL-6, IL-8, IL-9, IFN-γ, TNF, CCL2, IL-22	Prediction of the response to biologics therapy and mucosal healing (44–51)
	IL-2, IL-6	Disease relapse (52)
Non-coding RNA	miRNA	Diagnostic potential, classification, monitoring of disease activity, stricturing phenotype, glucocorticoids resistance (53)
	lncRNA	Diagnostic potential (54, 55)
Metabolomics		Potential of diagnosis and classification (56–60)
Proteomics		Potential of diagnosis, classification, differential diagnosis (61–64)
Galectins		Diagnostic potential (65, 66)
Vitamin D		Prediction of disease recurrence, hospitalizations, surgeries, response to anti-TNF-α therapy (67–70)
OSM		Diagnostic potential (71, 72)
ECM components	PIIINP, PICP, ITCP, fibronectin, laminin, TIMPs, COMP, HGFA	Fibrostenotic disease (73–82)
Growth factors	bFGF, YKL-40, VEGF	Fibrostenotic disease (83–86)

*IBD, inflammatory bowel disease; CD, Crohn's disease; UC, ulcerative colitis; pANCA, perinuclear anti-neutrophil cytoplasmic antibodies; ASCA, anti-Saccharomyces cerevisiae antibodies; anti-GP2, anti-glycoprotein 2 pancreatic antibodies; CUZD1, CUB and zona pellucida-like domains 1; anti-CHI3L1, anti-chitinase-3-like protein 1; anti-ACA, anti-cardiolipin; anti-PS/PT, anti-phosphatidylserine/prothrombin; anti-GM-CSF, anti-granulocyte macrophage colony-stimulating factor; ALCA, anti-laminaribioside carbohydrate antibodies; ACCA, anti-chitobioside carbohydrate antibodies; AMCA, anti-mannobioside carbohydrate antibodies; anti-OmpC, anti-Escherichia coli outer membrane porin C antibodies; anti-I2, anti-microbial sequence I2 antibodies; anti-CBir1, anti-flagellin CBir1 antibodies; anti-L, anti-laminarin antibodies; anti-C, anti-chitin antibodies; LL-37, cathelicidin; TFF3, trefoil factor 3; IL, interleukin; IFN-γ, interferon-γ; TNF, tumor necrosis factor; miRNA, microRNA; lncRNA, long non-coding RNA; PIIINP, procollagen III N-terminal propeptide; PICP, C-terminal propeptide of type I collagen; ITCP, C-terminal telopeptide of type I collagen; TIMPs, tissue inhibitors of metalloproteinases; COMP, cartilage oligomeric matrix protein; HGFA, hepatocyte growth factor activator; bFGF, basic fibroblast growth factor; YKL-40, human chitinase 3-like 1; VEGF, vascular endothelial growth factor*.

## Diagnosis and Classification of Inflammatory Bowel Disease

Previous studies had found out that detecting several particular antibodies against unequivocal antigen is a serologic characteristic of IBD patients (87). Serological antibodies, including autoantibodies and microbial antibodies, are produced on account of the excessive autoimmune responses, intestinal barrier injury, and losing immunological tolerance of bacterial antigens (88, 89). These antibodies have been proven to be useful biomarkers for diagnosis and classification of IBD (37). Recently, some serological antibodies have been discovered to have a clinical value in predicting disease activity or therapeutic response. These new findings will also be reviewed in this section.

### Serological Antibodies

#### Perinuclear Anti-neutrophil Cytoplasmic Antibodies

Perinuclear anti-neutrophil cytoplasmic antibodies (pANCA) are antibodies that react with lysosomal enzymes in the cytoplasm of neutrophils and monocytes. Serum pANCA have been widely studied and are accepted to be UC specific and thus can differentiate UC from CD (21). Although at present pANCA is relatively consistent in patients with UC, pANCA titers change with disease activity in UC patients (22). However, the sensitivity of pANCA in the evaluation of patients with suspected UC is rather low (90). pANCA are significantly increased in UC patients and in CD patients with “UC-like” features. Nearly 25% of CD patients with left-sided colitis identified endoscopically or histopathologically and with symptoms similar to UC present with increased levels of pANCA, which limits the utility of pANCA in the subclassification of IBD (91). Autoantibodies to neutrophil proteinase 3 (PR3), one of the ANCA, may be a useful serological marker for distinguishing IBD subset. The positive rate of PR3-ANCA in UC patients is 15–40%, whereas in CD patients, the positive rate is 0–10% (92).

#### Anti-saccharomyces Cerevisiae Antibodies

Anti-*Saccharomyces cerevisiae* antibodies (ASCA) are antibodies to the mannan protein of *S. cerevisiae*, which have high specificity but low sensitivity in identifying CD owing to a genetic susceptibility of CD patients. Study has shown that an increased titer of ASCA is associated with genes involved in bacterial sensing and autophagy (93). ASCA are also a risk marker for early disease onset, fibrostenosing, and internal-penetrating disease behavior (23). However, the expression of ASCA is relatively low in patients with isolated colonic CD (94). Moreover, it should be noted that the expression of ASCA varies in different ethnic populations: the prevalence and titers of ASCA are significantly lower in Asian CD patients than Caucasian CD patients (95).

Pairing pANCA and ASCA provides greater discriminatory ability than either alone. Joosens et al. performed a prospective, long-term study that enrolled 97 IBD-unclassified patients. Over 6 years, 31 (32%) patients received a definitive diagnosis. Eighty percent of patients who were ASCA+/pANCA– were diagnosed with CD, 100% of patients who were ASCA–/pANCA+ were diagnosed with UC (63.6%) or “UC-like” CD (36.4%), indicating that combined testing for pANCA and ASCA has high specificity (96). Reese et al. performed a meta-analysis of 60 studies and showed that the combination of ASCA+/pANCA– had a specificity of 92.8% and a sensitivity of 54.6% for CD, and pANCA+/ASCA– had a specificity of 94.3% and sensitivity of 51.3% for UC (97). The low sensitivity limits their clinical value in differentiating between CD and UC. Different from previous studies, a retrospective cross-sectional study including 2,550 patients with IBD in Australia recently showed that seropositivity of pANCA was found in more than 80% of patients and had no difference between patients with CD and UC. The combined use of pANCA and ASCA cannot differentiate between subtypes of IBD in this cohort (98). This discrepancy should be noted in clinical practice.

#### Antibodies Against Exocrine Pancreas (PABs)

The major antigens of PABs are glycoprotein 2 (GP2) and CUB and zona pellucida-like domains 1 (CUZD1) (92, 99). Anti-GP2 pancreatic (GP2) autoantibodies are promising serum markers for differentiating CD from UC. GP2 is a membrane-bound receptor located in microfold cells of intestinal Peyer's patches, which interacts with fimH-positive bacteria and mediates bacteria-specific mucosal immune responses (100). The GP2-rich M cells are abundant in the small intestine, whereas they are rare in the colon. The release of anti-GP2 antibodies is related to ileal inflammation, which may account for higher expression of GP2 in the serum as well as inflamed mucosa of CD patients as compared with patients with UC (101). Moreover, it is reported that patients with exclusively colonic CD presented significantly lower titers of anti-GP2 antibodies than did CD patients with ileum involvement (24, 25). Anti-major zymogen glycoprotein (MZGP2) antibodies can be detected using ELISA-based assays, which make it easier to detect than GP2 autoantibodies. Pavlidis et al. demonstrated that anti-MZGP2 antibodies were detected in 31% of CD patients and only 4% of UC patients and had a high specificity for CD (96%). Seropositivity of ASCA together with anti-MZGP2 antibodies had a 100% positive predictive value in discriminating CD from UC. High anti-GP2 levels were also found to be associated with early age of disease onset, extensive disease, ileo-colonic location, and longer disease duration (102, 103). Unlike GP2, biological function of CUZD1 is still unknown. Some researchers considered that CUZD1 might be involved in regulating the balance of immune response and immune tolerance (104). Both anti-GP2 and anti-CUZD1 are elevated in CD patients, compared with UC patients. Also, these antibodies were found to be associated with clinical phenotypes and disease behavior. Anti-CUZD1 positivity was associated with ileocolonic and perianal lesion, and anti-GP2 positivity was associated with stricturing behavior (26). Maria et al. found that time frame of surgery or development of perianal disease was associated with anti-GP2 or anti-CUZD1 positivity (105). But another study suggested that anti-CUZD1 positivity was not correlated with clinical outcome (106). However, the use of PABs in IBD diagnostic should be done with caution because PABs have been detected in many other diseases like refractory celiac disease (CeD), primary sclerosing cholangitis, and cholangiocarcinoma (107, 108).

#### Other Autoantibodies

Anti-granulocyte macrophage colony-stimulating factor (anti-GM-CSF) antibodies have also been shown to have higher concentrations in CD patients when compared with UC patients and healthy controls. Elevated levels of anti-GM-CSF antibodies are also associated with aggressive disease and ileal involvement in patients with CD (28). Nora et al. studied the antiphospholipid antibodies in CD and found that only anti-cardiolipin (anti-ACA) and anti-phosphatidylserine/prothrombin (anti-PS/PT) antibodies have significantly higher positive rate in patients with CD than UC and HC, whereas positivity of antiphospholipid antibodies has no clinical correlates (29). Lately, anti-chitinase-3-like protein 1 (anti-CHI3L1) and both IgG (IgA and sIgA) were found to have higher levels in patients with CD than in patients with UC and CeD (27). In addition, IgA and sIgA of anti-CHI3L1 have a higher diagnostic value in CD and are associated with complicated progression of CD (27). Another autoantibody, anti-goblet cell antibodies (GBA), a specific marker for UC, was positive in 11–28% of UC patients (109, 110).

#### Anti-microbial Antibodies

The levels of antibodies to the cell wall carbohydrate epitopes of bacteria, such as laminaribioside carbohydrate (ALCA), chitobioside (ACCA), and mannobioside carbohydrate (AMCA), are higher in patients with CD compared with patients with UC and healthy subjects (30). However, the combination of these antibodies and ASCA i not useful for the subclassification of IBD (87). Paul et al. demonstrated that a higher level of ACCA (>51 U/ml) and anti-laminarin (>31 U/ml) were significantly associated with steroid dependency of IBD patients. Moreover, they further defined that a severe CD outcome was link to a rising level of AMCA (>77 U/ml), ASCA (>63 U/ml), and ACCA (>50 U/ml), whereas a severe UC outcome was associated with an elevated level of AMCA (>52 U/ml) and ACCA (>25 U/ml) (31). ACCA are associated with a fibrostenosing or fistulizing behavior (111). Antibodies against the outer membrane protein C (anti-OmpC), microbial sequence I2 (anti-I2), and flagellin (anti-CBir1) have been reported to be more specific for CD than UC, suggesting that these antibodies may aid in differentiation between subtypes of IBD (32). Hamilton et al. reported that patients with repeated surgeries were more likely to be seropositive for anti-OmpC antibodies than are other patients (94% vs. 55%, ≥2 resections vs. <2 resections, *P* = 0.001) (33). A meta-analysis that studied four antibodies (ASCA, anti-OmpC, anti-I2, and anti-CBir1) showed that anti-OmpC antibodies had the highest specificity for needing surgery, and ASCA had the highest sensitivity for surgery (34). Anti-CBir1 and anti-I2 antibodies have been observed to be related to the stricturing behavior, longer disease duration, and early postoperative recurrence in patients with CD (33, 36, 37). Recently, a multicenter inception cohort study built a competing-risk model and showed that anti-CBir1 seropositivity was significantly associated with a stricturing and penetrating phenotype in pediatric CD (112). The other two anti-glycan antibodies against laminarin IgA (anti-L) and chitin (anti-C) showed high specificity for CD but had a low sensitivity. These two antibodies were found to be associated with penetrating behavior and the need for surgery (38).

### Circulating Non-coding RNAs

Non-coding RNAs (ncRNAs) are RNAs without protein coding potential and are important regulatory mediators transcribed from the genome and control gene expression at the RNA level, including microRNA (miRNA) and long ncRNA (lncRNA) (113). The aberrant expression of ncRNAs is often associated with several autoimmune diseases and malignant tumors (114). Recent studies have revealed their regulatory role in the pathogenesis of IBD. The expression profiles of ncRNAs from colon tissues and blood are different between IBD patients and healthy controls (115). Herein, we discuss the potential of circulating miRNA and lncRNA as biomarkers in the diagnosis of IBD (Table 2).

**Table 2:** Serum miRNAs proposed for IBD management.

**References**	**MiRNA**	**Association**	**Predictive value**
Iborra et al. (116)	MiR-188-5p, miR-877, miR-140-5p, miR-145, miR-18a, miR-128	Active CD	log_2_ FC(aCD/iCD) 1.47–3.00, *P* < 0.05
Wang et al. (117)	MiR-223	Disease activity of IBD	Correlation analysis of serum miR-223 with CDAI, SES-CD, UCEIS, Mayo score: *r* = 0.349–0.506, *P* < 0.05
Paraskevi et al. (118)	MiR-16, miR-23a, miR-29a, miR-106a, miR-107, miR-126, miR-191, miR-199a-5p, miR-200c, miR-362-3p, miR-532-3p	Diagnosis of CD	FC(CD/HC) 2.17–7.26, *P* < 0.05
Paraskevi et al. (118)	miR-16, miR-21, miR-28-5p, miR-151-5p, miR-155, miR-199a-5p	Diagnosis of UC	FC(UC/HC) 2.98–7.82, *P* < 0.05
Wu et al. (119)	MiR-199a-5p, miR-362-3p, miR-340,-532-3p, miRplus-1271	Diagnosis of IBD	FD(aCD/HC), FD(aUC/HC), sens, spec: ND, *P* < 0.05
Zahm et al. (120)	MiR-16, miR-484, miR-30e, miR-106a, miR-195, miR-20a, miR-21, miR-140, let-7b, miR-192, miR-93	Diagnosis of pediatric CD	AUC: 0.821–0.917, sens: 69.57–82.61%, spec: 75.00–100%, *P* < 0.05
Schonauen et al. (121)	MiR-16, miR-21, miR-223	Diagnosis of IBD	FC(IBD/HC) _miR−16_: 2.9-fold, FC_miR−21_:2.7, FC_miR−223_: 3.8, *P* < 0.05
Krissansen et al. (122)	MiR-595, miR-1246	Active IBD	FC(aCD/iCD)_miR−1246_: 5.4-fold, FC(aUC/iUC)_miR−1246_: 3.45; FC (aCD/iCD) _miR−595_: 1.9, FC (aUC/iUC) _miR−595_: 1.8 (*P* < 0.05)
Chen et al. (123)	MiR-146b-5p	Endoscopically active disease of IBD	CD classifier: AUC 0.869, sens: 84.91%, spec: 84.62%, *P* < 0.001
Nijhuis et al. (124)	MiR-29a	Stricturing CD	FD(SCD/NSCD), sens, spec: ND, *P* = 0.049
Lewis et al. (125)	MiR-19a-3p, miR-19b-3p	Stricturing CD	FD(SCD/NSCD)>2-fold, *P* < 0.01

*IBD, inflammatory bowel disease; CD, Crohn's disease; UC, ulcerative colitis; FC, fold change; CDAI, Crohn's disease activity index; SES-CD, simple endoscopic score for CD; UCEIS, Ulcerative Colitis Endoscopic Index of Severity score; aCD, active CD; iCD, inactive CD; aUC, active UC; iUC, inactive UC; HC, healthy controls; r, Spearman rank correlation coefficient; ND, no data available; SCD, stricturing CD; NSCD, non-stricturing CD*.

#### MicroRNAs

MiRNAs are short ncRNAs that negatively regulate gene expression at the post-transcriptional level. MiRNAs are assembled in RNA-induced silencing complex (RISC), which prevents translation or degrades mRNA by binding the 3′-untranslated region of mRNA (126). They hold promise as non-invasive biomarkers of disease activity because of their stability in circulation due to their short length (about 18–24 nucleotides). Recent studies have shown that miRNAs mediate inflammatory responses and intestinal barrier function in the pathogenesis of IBD. MiR-192 was shown to be downregulated in the colonic mucosa of patients with active UC. Further study demonstrated that miR-192 was an important mediator for inhibition of the expression of a pro-inflammatory chemokine, macrophage inflammatory peptide 2a (116). Our previous study showed that miR-223 was increased in inflamed colonic mucosa of IBD patients. MiR-223 targets claudin-8, a crucial protein of the tight junctions of intestinal mucosa, through the IL-23 pathway and impairs intestinal barrier function (127). The level of miR-223 is significantly increased in the circulation and correlates closely with disease activity in patients with CD and UC (117). Moreover, miRNAs also play important roles in endoplasmic reticulum stress and gut microbiota interactions in IBD (128–130).

Since Wu et al. first reported 11 differentially expressed miRNAs in the colon tissues of patients with active UC, several groups have analyzed miRNA profiles in tissues of patients with IBD using microarray methods (131, 132). Serum miR-200c and miR-155 have been proven to be involved in the pathogenesis of IBD and over-expressed in tissues of IBD patients (133, 134). Moein et al. highlighted that colonic miRNA (miR-31, miR-24, and miR-126) may be a potential marker in IBD diagnosis and classification in a recent review (135). However, still, few studies have examined serum miRNAs in patients with IBD. Paraskevi et al. identified 11 serum miRNAs that are increased in patients with CD and six serum miRNAs (miR-16, miR-21, miR-28-5p, miR-151-5p, miR-155, and miR-199a-5p) that are increased in patients with UC as compared with healthy controls (118). Wu et al. identified seven altered serum miRNAs in CD patients and 12 serum miRNAs in UC patients (119). Zahm et al. reported 11 CD-associated serum miRNAs, which may be valuable in the diagnosis of pediatric CD with sensitivities between 71 and 83% and specificities between 75 and 100%. Patients with CeD were included as patient controls to investigate the specificity of these candidate miRNAs, and no significant difference was observed between patient controls and healthy controls (120). MiR-16, miR-106a, and miR-532-3p are overlapping miRNAs in the aforementioned studies. Serum miRNAs may also be valuable in IBD classification. Schonauen et al. reported serum concentrations of miR-16, miR-21, and miR-223 were higher in IBD patients than healthy controls, and levels were higher in CD compared with UC (121). It is noted that increased expressions of miR-21 were also observed in subsets of macrophages and T cells in mucosal tissue of UC patients compared with CD patients (136).

#### Long Non-coding RNAs

LncRNAs are non-coding RNAs involved in the regulation of various intracellular processes and have a length of more than 200 nucleotides (137). Studies on lncRNAs in IBD have increased in the past decade and have found dysregulated expression in blood samples and biopsy tissue. LncRNAs have been proven to play important roles in IBD pathogenesis, including regulation of the intestinal epithelial barrier, cell apoptosis, and various immune system processes (138, 139). There is growing evidence that lncRNAs may be promising diagnostic markers of various cancers, cardiovascular disease, and autoimmune disorders because they are relatively stable and simple to detect (140–142). Chen et al. evaluated circulating lncRNAs levels of CD patients using microarray screening and qRT-PCR. The lncRNA GUSBP2 had the highest upregulation, and the lncRNA AF113016 the greatest downregulation. The expressions of eight circulating lncRNAs (NR_033913, NR_038218, NR_036512, NR_049759, NR_033951, NR_045408, NR_038377, and NR_039976) were changed as compared with those of healthy controls, indicating they may hold diagnostic potential in patients with CD (54). Wang et al. selected three differentially expressed lncRNAs (KIF9-AS1, LINC01272, and DIO3OS) identified in prior studies, and they evaluated their diagnostic value in IBD. They reported that KIF9-AS1 and LINC01272 were upregulated and DIO3OS was downregulated in patients with IBD as compared with healthy controls. The area under the receiver operating characteristic (ROC) curve (AUC) of KIF9-AS1 is 0.811 for discriminating CD patients from healthy controls and 0.872 for discriminating UC patients from controls (55). However, to date, few studies have examined non-invasive blood-based lncRNA profiles with respect to IBD. More studies are needed to confirm previous results, identify new lncRNAs, and systematically evaluate their diagnostic value.

### Metabolomics

It has been accepted that the development of IBD is associated with the interaction between host and gut microbiota. Because the metabolome of a patient derives from host metabolism, and part of gut microbiota metabolism is influenced by changes of the environment, there is increasing interest in using metabolomics to elucidate the pathogenic mechanisms of IBD and improve diagnosis (143). Williams et al. performed serum metabolic profiling in patients with CD, patients with UC, and healthy controls using ^1^H nuclear magnetic resonance (NMR) spectroscopy. Using partial least squares discriminant analysis with orthogonal signal correction, the authors showed significant differences in lipid and choline metabolism between CD and UC (56).

Hisamatsu et al. studied plasma amino acid profiles in IBD. A multivariate index built with the plasma aminogram, including histidine and tryptophan, exhibited significant accuracy in discriminating CD and UC (57). Another group using gas chromatography/mass spectrometry (GC/MS) reported that serum profiles of amino acids and tricarboxylic acid (TCA) cycle-related molecules were different between UC patients and healthy controls, and between UC and CD patients (58).

Recently, Scoville et al. studied serum metabolite profiles using ultra-high-performance liquid chromatography/mass spectrometry (UPLC-MS/MS) and identified 173 altered metabolites, including lipids, amino acids, and TCA metabolites in IBD patients compared with healthy subjects. Whereas, 286 serum metabolites were found to be significantly changed in CD patients compared with health subjects, only five metabolites were found to be decreased in patients with UC. Fatty acid, acylcarnitine metabolite, sphingolipid, and bile acid metabolism were significantly different in CD patients compared with UC patients and healthy controls (59). Serum metabolite profiles are also different in pediatric CD and UC patients (60). Taken together, these studies indicate that serum metabolic profiling holds promise in differentiating IBD subtypes.

### Proteomics

The proteome reflects the interaction between genetic susceptibility and environmental factors and can be regarded as the markers of disease. Several groups have evaluated the value of serum protein profiling to improve the management of IBD. Meuwis et al. evaluated serum protein profiles in four groups (30 CD patients, 30 UC patients, 30 inflammatory controls, and 30 healthy controls) using surface-enhanced laser desorption/ionization–time of flight–mass spectrometer (SELDI-TOF-MS). Four serum proteins associated with acute-phase inflammation (platelet aggregation factor 4, haptoglobin a2, fibrinopeptide A, and myeloid-related protein 8) were identified and showed diagnostic value of IBD with sensitivities and specificities higher than 80% (61). Whereas, Meuwis recruited patients with asthma and rheumatoid arthritis as inflammatory controls, Zhang et al. investigated serum proteomic profiling in differentiating intestinal tuberculosis (ITB) and IBD. They applied 10 mostly differentially expressed proteins to build a diagnostic model and a differential diagnostic model using support vector machine. The diagnostic model composed of four proteins was able to distinguish CD patients from healthy controls, with a specificity of 96.7% and a sensitivity of 96.7%. A differential diagnosis model containing three proteins could differentiate CD patients from ITB patients, with a specificity and sensitivity of 76.2 and 80.0%, respectively (62).

Genome-wide association studies (GWASs) have identified nearly 200 susceptibility genes associated with IBD and greatly improved our understanding of IBD etiology (63). Drobin et al. detected serum protein profiles encoded at 163 IBD risk loci from 49 CD patients, 51 UC patients, and 50 healthy controls and evaluated differentially expressed proteins in another independent group with 64 IBD patients. Thirteen proteins related to cytokine signaling, immune-metabolic regulation, and immune cell activation were differentially expressed in IBD patients. Three serum proteins (LACC1, SAA, and LNPEP) and two proteins (CNTF and LPXN) were specifically altered in CD patients and UC patients, respectively, compared with healthy individuals (64).

### Oncostatin M

Oncostatin M (OSM), a member of IL-6 cytokine family, is highly and consistently expressed in IBD patients, in both inflamed mucosa and blood. West et al. first demonstrate the elevated serum level of OSM in active IBD patients, but a serum OSM level at diagnosis cannot predict the disease outcome in IBD patients (71). Recently, Verstockt et al. found out that first-degree relatives in multiple-affected IBD families have increased serum OSM levels than do matched control families, with levels similar to those of IBD patients (72). These findings indicated that the serum level of OSM could be a diagnostic biomarker of IBD patients. Unlike the colonic OSM level, the serum level of OSM is not a predictive biomarker for anti-TNF responsiveness and disease outcome (71). As a new discovery of IBD biomarker, OSM has gained much attention in the past few years, but its accuracy and potential value in IBD need to be further evaluated.

### Serum Galectins

Galectins are a family of mammalian galactosidase-binding proteins that are associated with malignancies and inflammation conditions. Elevated levels of serum galectins were detected in colon cancer patients compared with healthy people. Galectins are involved in disease mechanisms of IBD by mediating apoptosis of T lymphocytes and NF-kappa B signaling. Serum galectin-3 were higher in patients with IBD associated with emerging positivity of CD14+ cells (65). Recently, Yu et al. reported that the serum levels of galectin-1 and galectin-3 were significantly higher in IBD patients compared with healthy controls. However, no significant difference was found between patients with active disease and patients in remission stage (66).

## Surveillance of Disease Activity

### C-Reactive Protein

C-reactive protein (CRP) is an acute-phase reactant, consisting of five identical non-covalently bound monomers. It is produced by hepatocytes in response to stimulation from inflammatory cytokines such as interleukin-1 (IL-1), IL-1β, and tumor necrosis factor-alpha (TNF-α) and has a relatively short half-life of about 19 h. Under normal conditions, the serum level is low (<1 mg/L) but increases rapidly more than 1,000-fold during acute inflammation (144–146). CRP is the most widely used serum indicator of inflammation in IBD. Increased levels of CRP help differentiate mucosal active disease from quiescent IBD. CRP level <10 mg/l indicates remission stage of IBD (40). Endoscopic disease activity correlates well with the serum CRP level (41).

Clinical symptoms of CD and an elevated CRP level are consistent with disease recurrence (147). However, CRP is not disease specific, and elevated levels occur in non-IBD enteritis, inflammatory disorders not related to the gastrointestinal tract, tissue damage, diabetes, malignancies, and cardiovascular disease (148–152). The sensitivity of CRP measurement is also limited with respect to IBD; normal CRP levels can be seen in patients with active IBD (153). CRP levels may also be normal in asymptomatic patients with mild mucosal lesions, especially isolated involvement of the ileum. Up to 28% children of CD and 42% pediatric UC were observed normal CRP levels (154). Suk et al. found that the level of CRP differed among individuals with the same inflammatory conditions owing to genetic factors (155). Age, sex, and body mass index also affect the serum levels of CRP (156). Although CRP levels correlate well with CD and patients present with higher levels than those with UC, it cannot be used to distinguish CD from UC (157). In pediatric IBD, serum levels of CRP cannot distinguish active disease from quiescent disease and cannot be used to evaluate disease activity in patients treated with systemic GCs (38).

### Serum MicroRNAs

Circulating miRNAs are mainly derived from exosomes of different cell types that actively secrete them into the circulation, or are released passively from apoptotic and necrotic cells (158). It is assumed that circulating miRNAs derived from exosomes are secreted in the course of inflammatory signaling and reflect miRNA expression changes of inflamed gut mucosa (118, 120). Nevertheless, circulating miRNAs appear to be inconsistent with tissue miRNAs in patients with active CD and active UC (116). In order to identify disease recurrence, further studies are needed to identify serum miRNAs, which correlate well with disease activity and can distinguish active disease from quiescent disease. Iborra et al. reported two circulating miRNAs (miR-188-5p and miR-877) with higher expression and four miRNAs (miR-140-5p, miR-145, miR-18a, and miR-128) with lower expression in patients with active CD compared with those with quiescent CD (116). Serum miR-595 and miR-1246 were significantly increased in the serum of active colonic CD and UC compared with inactive diseases (122). Recently, we found that serum expression of miR-146b-5p was elevated in patients with IBD and changed according to disease activity. A CD miRNA classifier was built that combined the serum miR-146b-5p level and platelet count, and the classifier exhibited higher accuracy in identifying mucosal active CD with a sensitivity of 84.9% and a specificity of 84.6% than using serum miR-146b-5p alone, or CRP alone and thus may be of value for monitoring disease activity (123). Larger cohort studies to verify these results and identify more promising miRNAs for monitoring IBD may be warranted.

### Miscellaneous Indicators

A number of novel serum indicators, which may be helpful in identifying disease activity of IBD, were reported recently. Fecal calprotectin rejects the migration of neutrophils through the inflamed bowel wall to the mucosa, which has been shown to be closely related to intestinal mucosal inflammation. Fecal calprotectin predicts mucosal active disease with a >90% positive predictive value in patients with CD (159). Recently, Suarez et al. found that serum calprotectin had higher AUC for disease activity than CRP, erythrocyte sedimentation rate, hemoglobin, and platelets (160). Oxidative stress plays an important role in IBD pathogenesis. Serum free thiols (R-SH) decreased during systemic oxidative stress owing to consumption of oxidation reaction. Patients with IBD were found to have lower serum R-SH levels than healthy individuals. Serum R-SH levels distinguish moderate-to-severe disease activity from mild disease with higher accuracy than fecal calprotectin (AUC 0.87 vs. 0.76, *P* < 0.05) (161).

Serum cathelicidin (LL-37) levels were negatively correlated with disease activity of IBD patients (partial Mayo scores of UC and Harvey–Bradshaw indices of CD). Patients with higher initial levels of serum LL-37 showed better prognosis than did the patients with low initial cathelicidin levels. Low LL-37 levels predicted stricture disease in patients with CD (42). Trefoil factor 3 (TFF3) is mainly secreted by goblet cells in gastrointestinal tract and protects the epithelial barrier function of mucosa. Higher levels of serum TFF3 were detected in patients with active IBD than patients with inactive IBD (*P* < 0.001). Serum TFF3 levels correlated closely with Ulcerative Colitis Endoscopic Index of Severity (UCEIS) (*r* = 0.662, *P* < 0.001) (43).

Xu et al. reported higher expression of serum fibrinogen in patients with active CD compared with quiescent CD (*P* = 0.018) (162). Margarita et al. evaluated 27 protein biomarkers including serum cytokine, chemokine, and growth factor in IBD patients with different endoscopic activities. Patients with endoscopically active disease showed higher serum levels of granulocyte colony-stimulating factor (G-CSF) (*P* = 0.04), IL-1 receptor antagonist (IL-1Ra) (*P* = 0.04), and platelet-derived growth factor BB (*P* = 0.02). Increased serum expression of interferon-induced protein 10 was associated with extraintestinal manifestations (arthritis) of IBD (*P* = 0.041) (163).

## Biomarkers Predicting Clinical Response

### Pro-Inflammatory Cytokines

Pro-inflammatory cytokines, which mediate interaction between immune cells and non-immune cells, contribute to the inflammatory status of the intestine. Accumulating evidence suggests that cytokines may be useful for predicting the response to anti-TNF therapy (164). CD patients with low serum baseline concentrations of IL-1β (<0.64 pg/ml) were more likely to achieve infliximab (IFX) response (44). Billiet et al. found that primary IFX therapy response was associated with lower serum IL-8 levels and higher concentrations of albumin at week 6. Serum levels of IFN-γ and IL-6, which decrease at week 2 and week 6 relative to baseline levels during IFX induction, can predict the primary response to treatment. The TNF/CRP ratio may predict lack of response to IFX therapy at week 14 [odds ratio (OR) = 2.8, 95% confidence interval (CI): 1.4–5.5] (45). Moreover, serum IL-9 concentrations have been shown to be higher in active CD patients than in healthy controls (22.0 vs. 6.3 pg/ml) and differ between patients with moderate-to-severe CD (29.1 pg/ml) and patients with mild disease (12.9 pg/ml). Decreased levels of serum IL-9 at week 14 compared with baseline levels are predictive for mucosal healing and clinical remission at week 30 (AUC = 0.752 and 0.803, respectively) (46). In patients with UC, non-responders have been found to have lower TNF and IL-1β levels, which is due to the impaired innate responses to all TLR agonists and is associated with the number of plasmacytoid dendritic cells and CD4+ regulatory T cells (47). Moreover, a decrease in serum concentration of CCL2 between baseline and week 2 is associated with clinical response (48). Recently, Obraztsov et al. explored a better way to predict response to anti-TNF therapy with cytokines. A predictive model was built combining seven cytokines—TNF-α, IL-12, IL-8, IL-2, IL-5, IL-1β, and IFN-γ–using the Fisher linear discriminant analysis, which was able to discriminate between responders and non-responders with a sensitivity of 84.2% and a specificity of 93.3% (49).

Apart from indicators for predicting response to anti-TNF therapy, multiple biomarkers were identified of response to new biologic therapies for IBD. MEDI2070 is a new biological agent for IBD, a monoclonal antibody that inhibits IL-23 binding to its receptor. Bruce et al. evaluated the efficacy of MEDI2070 in patients with moderate-to-severe CD who had failed to respond to TNF antagonists in a phase 2a trial and found that patients treated with MEDI2070 achieved clinical improvement compared with patients receiving placebo. Patients with higher serum IL-22 levels at baseline were more likely to respond to MEDI2070 (50). Recently, Bertani et al. evaluated the predictive value of pro-inflammatory cytokines of clinical remission in UC patients treated with vedolizumab. Higher baseline serum IL-8 levels and significant decreased levels of IL-6 and IL-8 over the first 6 weeks were associated with clinical remission. A nomogram was built using these predictors presenting a sensitivity of 82% and a specificity of 90% to predict clinical remission and a sensitivity of 83% and specificity of 87% to predict mucosal healing (51). Colonic integrin αe and granzyme A level can predict response to etrolizumab (165). The predictive value of these biomarkers in response to new therapies still needs further investigations.

### Monitor Test

Recently, a new serologic test, known as the monitor test or the mucosal healing index (MHI), comprising 13 serum proteins [carcinoembryonic antigen-related cell adhesion molecule, vascular cell adhesion molecule, CRP, serum amyloid A, angiopoietin-1, angiopoietin-2, matrix metalloproteinase (MMP)-1, MMP-2, MMP-3, MMP-9, extracellular matrix (ECM) metalloproteinase inducer, transforming growth factor (TGF)-α, and IL-7], was established using multiple logistic regression models to predict mucosal healing in patients with CD. The computed score reflects endoscopic disease activity. The validation cohort included patients who received anti-TNF therapy in the TAILORIX trial (Study Investigating Tailored Treatment With Infliximab for Active Crohn's Disease) and showed an accuracy of 90%, a positive predictive value of 87%, and a negative predictive value of 92% for detecting endoscopic lesions in CD patients (166). Another group evaluated the utility of MHI in disease recurrence after surgeries and found that MHI significantly correlated with the Rutgeerts Score 6 and 18 months after surgeries. An ROC curve showed that a cut-off value of <20 excludes disease recurrence with an 87.5% sensitivity, and a cut-off value ≥ 40 predicts severe recurrence of CD with a specificity of 93% (167).

In UC patients who received anti-TNF therapies, De Bruyn et al. developed an Ulcerative Colitis Response Index (UCRI) with serum neutrophil-related markers (CRP, CHI3L1, LL-37, and neutrophil count) to predict mucosal healing. These neutrophil-related markers were decreased significantly in patients who responded to treatment and lower in healers than non-healers. UCRI can accurately detected mucosal healing with AUCs higher than 0.79 in both IFX-treated and adalimumab-treated groups (168).

### Vitamin D

Vitamin D (25-hydroxyvitamin D; [25(OH)D]) is an immune modulator of the innate and adaptive immune systems, and deficiency in IBD patients is associated with an increased risk of disease recurrence, hospitalizations, and surgeries (67). CD patients show high prevalence of vitamin D deficiency and have significantly lower vitamin D levels than do patients with irritable bowel syndrome (169). Serum vitamin D level is negatively correlated with disease activity in CD patients (170). Patients with a low vitamin D level (<30 ng/ml) are more likely to stop anti-TNF therapy early owing to a loss of response (HR = 3.49, 95% CI: 1.34–9.09) (68). Santos-Antunes et al. demonstrated that extreme vitamin D deficiency (<20 ng/ml) was associated with serum antinuclear antibody (ANA) positivity, anti-TNF failure, and adverse events (OR = 20.11, 95% CI: 2.10–192.39) (69). However, Reich et al. found that CD patients with a low serum vitamin D level had a higher clinical remission rate at 14 weeks than patients with normal vitamin D levels (80% vs. 23%, *P* = 0.007) (70).

### Miscellaneous

A multicenter retrospective study showed that CRP ≥ 3 mg/dl (OR = 4.77, 95% CI: 1.43–15.94) predicted a good response to IFX (171). However, Eriksson et al. reported that the elevated CRP at baseline in patients treated with vedolizumab were associated with higher risk of loss of response (HR: 2.22, 95% CI: 1.10–4.35) (172). Reinisch et al., who conducted a *post hoc* analysis of the ACCENT I study, found that the high baseline CRP level and low CRP level at week 14 were independently associated with maintained response to IFX therapy (173). Another group showed that a greater change in the CRP level between baseline and week 14 may predict a sustained response to IFX therapy (174). Another study showed that a normalized CRP level at week 12 predicted medium-term clinical remission and mucosal healing during anti-TNF-α therapy in CD patients (175). High CRP levels at IFX initiation (>10 mg/dl) are associated with colectomy in patients with UC (HR = 5.11, 95% CI: 1.77–14.76) (176). Morita et al. demonstrated that patients who responded to anti-TNF therapies had significantly lower CRP levels at 2 weeks than had non-responders. Moreover, UC patients with higher serum albumin concentrations were more likely to achieve clinical response at 8 weeks, as well as mucosal healing (177). Another group also reported higher serum albumin levels before induction therapy in responders than in non-responders (178). Schoenefuss et al. explored the predictive value of serum albumin and γ-globulin for secondary loss of response to anti-TNF therapy. Similarly, patients with low levels of serum albumin and high levels of γ-globulin predicted secondary loss of response (179).

As previously mentioned, pANCA holds promise for the subclassification of IBD. Negative pANCA status has been shown to be associated with a positive clinical response to IFX in patients with moderate-to-severe UC (180). In a pooled analysis, patients with negative pANCA had twice the clinical response rate of patients with positive pANCA (OR = 1.87, 95% CI: 1.02–3.41) (181). Patients with a baseline pANCA+/ASCA– serotype have been shown to have a lower early response rate to IFX therapy (55% vs. 76%; OR = 0.40, 95% CI: 0.16–0.99) (182).

On the other hand, inconsistent results have been shown in the associations of other antibodies and clinical response. While Santos-Antunes et al. reported a relation between elevated ANA level and treatment failure (*P* = 0.008) (69), Kiss et al. found no association between ANA seropositivity and treatment efficacy or adverse outcomes; instead, the authors found that dsDNA positivity maybe associated with a lack of response of anti-TNF therapy. Additionally, low levels of serum anti-IFI16 IgG before IFX induction have been shown to indicate the clinical response to treatment (OR = 0.14, 95% CI: 0.03–76): increased titers of anti-IFI16 antibodies were found in most patients who achieved a clinical response or remission (*P* = 0.05) (39). Anti-OmpC seropositivity has been associated with poor clinical response (OR = 0.14, 95% CI: 0.03–0.60) and cessation of anti-TNF therapy (HR = 2.20, 95% CI: 1.10–4.70) at 1 year (35).

Bjerrum et al. investigated whether serum metabolite profiles can predict response to IFX. Although no metabolites examined predicted treatment response, circulating proatherogenic lipid profiles were found in IBD patients receiving IFX induction, which may be the reason IBD patients have an elevated risk of developing cardiovascular disease (183). Luo et al. found that serum miRNAs may serve as indicator for GC resistance in patients with UC. Eight serum miRNAs expression were downregulated in GC-resistant patients with AUCs higher than 0.85, specificities between 73.00 and 97.30%, and sensitivities between 66.70 and 97.40% (53).

## Prognostic Prediction

Previous studies found that high serum levels of IL-2 and IL-6 had a predictive value for a relapse within 12 months (52). Another study found that patients who were positive for anti-CBir1, anti-OmpC, anti-A4-Fla2, and anti-Fla-X antibodies before surgery had a higher risk of recurrence within 18 months (33). The positivity of CD associated anti-microbial antibody can predict future onset of CD. Choung et al. reported that 65% of patients had at least one antibody seropositivity before a diagnosis of CD (184). Moreover, as previously described, several antibodies predict the risk of complicated disease behavior, such as stricturing or penetrating behavior. Patients with long-term CD often develop intestinal stenosis and subsequent obstruction due to transmural inflammation of intestine followed by tissue remodeling and transmural fibrogenesis. Although fibrostenosis is less common in UC, accumulating evidence has indicated that there is fibrotic rearrangement of the colonic wall even in short-lasting UC (185). Herein, we discuss biomarkers for intestinal fibrosis of IBD patients.

### Serum Biomarkers Indicating Intestinal Fibrosis

#### Extracellular Matrix Components

The fibrotic changes of the intestinal wall are due to hypertrophy of smooth muscles and excessive accumulation of ECM components, including fibronectin, laminin, collagen, procollagen III N-terminal propeptide (PIIINP), C-terminal propeptide of type I collagen (PICP), and C-terminal telopeptide of type I collagen (ITCP) (186). Plasma fibronectin has a significant but weak correlation with disease activity in CD patients. Higher plasma fibronectin levels have been observed in patients with strictures requiring operation; however, although plasma concentrations are significantly reduced after surgical intervention, they do not predict the development of postsurgery strictures (73, 74). Serum laminin levels are increased, whereas serum levels of collagen IV are decreased in IBD patients as compared with healthy controls. However, neither of them is correlated with localization or disease behavior (75).

The imbalance between MMPs and tissue inhibitors of metalloproteinases (TIMPs) plays an important role in fibrogenesis of the intestinal wall. Serum levels of MMP-9, which are involved in ECM degradation and angiogenesis, are significantly higher in active IBD than inactive disease and are correlated with disease activity (76). Lower serum TIMP-4 levels have been found in IBD patients compared with healthy controls, whereas concentrations of serum TIMP-1 were higher in patients with CD and UC than healthy controls and also higher in active IBD compared with inactive disease and thus are promising markers for the diagnosis of IBD and for disease monitoring (77). Additionally, reduced levels of serum TIMP-2 at 14 weeks from baseline predicted long-term remission and good prognosis of patients who accepted anti-TNF therapy (78).

Simone et al. reported that serum levels of PIIINP were higher in patients with stricturing CD and that levels were significantly reduced at 6 months after resection of the diseased intestine (79). The enhanced liver fibrosis (ELF) score, which includes serum TIMP-1, PIIINP, and hyaluronic acid levels, is used to assess liver fibrosis and may be useful for distinguishing stricturing CD from non-stricturing disease (80). A recent study evaluated plasma concentrations of collagen type III alpha 1 chain (COL3A1, also called PIIINP), cartilage oligomeric matrix protein (COMP), and colony-stimulating factor 2 antibodies (anti-CSF2 antibodies) at baseline in pediatric CD patients and followed up the patients for 36 months. Patients with higher levels of COL3A1 were more likely to develop strictures. A combination of baseline levels COL3A1 and anti-CSF2 antibodies identified patients with stricturing behavior from those with only inflammation with high accuracy (AUC = 0.80, 95% CI: 0.71–0.89). Median baseline plasma COMP level was not significantly different between groups (81). On the other hand, another group studying adult CD patients demonstrated that serum COMP concentrations (431.7 ± 112.7 vs. 348.7 ± 90.5 ng/ml, *P* = 0.012) and hepatocyte growth factor activator (HGFA) concentrations (152.7 ± 66.5 vs. 107.1 ± 38.7 ng/ml, *P* = 0.031) were elevated in patients with fibrostenotic disease compared with those with inflammatory disease. A significant reduction of serum HGFA levels was observed after resection of the affected intestinal segment (152.7 ± 66.5 vs. 107.1 ± 38.7 ng/ml, *P* = 0.015), but COMP levels did not differ between groups (82).

#### Growth Factors

Several growth factors play crucial roles in development of fibrostenosis. Whether or not their serum concentrations can be possible biomarkers of intestinal fibrosis in IBD has been studied. Serum concentrations of basic fibroblast growth factor (bFGF), which promotes tissue healing by regulating fibroblast proliferation, are higher in CD patients with strictures than in healthy controls and patients with inflammatory or fistulizing phenotypes. Serum vascular endothelial growth factor (VEGF) is increased in stricturing CD and correlated with intramural blood flow, making it a possible marker of angiogenesis (83). Additionally, high serum VEGF levels at baseline are associated with a poor response to anti-TNF-alpha therapy (AUC = 0.8): patients who achieve clinical remission at week 14 exhibit a reduction of serum VEGF levels. Human chitinase-3-like 1 (also known as YKL-40) is secreted by macrophages and neutrophils and promotes myofibroblasts to secrete collagen. Serum YKL-40 levels are increased in CD patients as compared with healthy controls and are higher in patients with a stricturing phenotype than in those without strictures (84). However, a different study found different results when comparing patients with or without strictures (85). Moreover, serum levels of YKL-40 are correlated with disease activity in UC patients, and patients with severe active disease have significantly higher levels than those with inactive UC (86).

#### Serum MiRNAs

The value of differentially expressed serum miRNAs profiles as biomarkers for the diagnosis and monitoring IBD has been widely studied. However, there is relatively limited knowledge regarding the role of miRNAs as fibrogenic modulators in IBD, and serum biomarkers of intestinal fibrosis. The most extensively studied miRNAs with respect to the pathogenesis of intestinal fibrosis are the miR-200 family and miR-29 family. Researches showed that miR-200b may inhibit epithelial-to-mesenchymal transition (EMT) through targeting zinc finger E-box-binding homeobox 1 (ZEB1) and ZEB2, which inhibit intestinal fibrosis. Serum levels of miR-200b are overexpressed in CD patients with a stricturing phenotype as compared with CD patients with other phenotypes, indicating the potential of serum miR-200b as a marker of fibrosis in patients with CD (187). Nijhuis et al. found that the miR-29 family (miR-29a, miR-29b, and miR-29c) were downregulated in the mucosa of strictured intestine from patients with stricturing CD as compared with that of non-strictured areas from patients with a non-stricturing phenotype. Similarly, low expression of miR-29 has been reported in renal, cardiac, and hepatic fibrosis (188). Other studies have shown that TGF-β induces the expression of collagen I and III through the suppression of miR-29. Moreover, serum expression of miR-29 is significantly lower in CD patients with stricturing behavior than in patients without strictures (124). Serum expressions of miR-19a-3p and miR-19b-3p are lower in patients with stricturing CD relative to patients with non-stricturing CD. A study has found that patients who develop a stricturing phenotype 4 years later tended to have lower expression of miR-19a-3p and miR-19b-3p (125).

## Conclusion

Because correct IBD management is important with respect to disease prognosis, extensive investigations of non-invasive serum biomarkers have been undertaken to find markers that are useful for disease diagnosis, subclassification, monitoring disease activity, and prediction of treatment outcomes and complications. Despite a great deal of study, current IBD biomarkers are far from ideal. Because individual biomarkers lack specificity or sensitivity, the combination of different biomarkers such as CD classifier, MHI, and UCRI may enhance the effectiveness in evaluating disease course. Further studies are required to identify new biomarkers that have low cost and improved availability. More attention should be paid to predicting complicated disease before disease progression and assessing the risks of re-hospitalization and postoperative recurrence. It should be noted that the methods for identifying new biomarkers and clinical trial endpoints should be rigorous and standardized. The assessment of disease activity and response to therapies needs to be objective. Newly discovered markers should be confirmed in multicenter international collaborations before they are applied to clinical practice.

## Author Contributions

The guarantor of the article is SZ. SZ, ZZ, and MC designed the study. SZ, PC, and GZ wrote and revised the manuscript. GZ, JL, and LL revised the important intellectual content of the manuscript. All authors approved the final version.

## Conflict of Interest

The authors declare that the research was conducted in the absence of any commercial or financial relationships that could be construed as a potential conflict of interest.
